# Upper Digestive Bleeding in Atrial Fibrillation: Evaluation of Gastroscopy Prior to Oral Anticoagulant Therapy (GOAT), Prospective, Randomized, Double Blind Study on a Community Population

**DOI:** 10.4021/cr37w

**Published:** 2011-03-25

**Authors:** Alfonso Lagi, Simona Spini, Elisa Meucci, Alessandro Cartei, Simone Cencetti

**Affiliations:** a Internal Medicine Department, Hospital of Santa Maria Nuova, Florence, Italy; b Emergency Department, Hospital of Santa Maria Nuova, Florence, Italy

**Keywords:** Atrial fibrillation, Risk factors, Cohort study, Anticoagulation, Digestive hemorrhage

## Abstract

**Background:**

The aim of this study was to investigate the incidence of digestive hemorrhages in patients with non-valvular atrial fibrillation (NVAF), scheduled for oral anticoagulant therapy.

**Methods:**

We conducted, over 24 months, a prospective, randomized, population-based observational study on consecutive patients with recurrent paroxysmal, persistent, or permanent NVAF, scheduled for oral anticoagulant therapy. The study initially included 268 patients with NVAF (162 males and 106 females) with a mean age of 74 years (range 42-86 years). Patients were split into two groups: those undergoing preventive Esophago-Gastro-Duodenoscopy (EGD) (Group A) and those who did not (Group B). All patients positive by EGD underwent medical treatment and subsequent 30-day endoscopic controls showed complete healing. The primary outcome of the study was to determine if previous EGD in patients with NVAF resulted in a low risk of bleeding during oral anticoagulant therapy. The two groups were comparable for most variables.

**Results:**

Significant differences were found between groups for the incidence of antiarrhythmic drugs and for early hemorrhage (*P* <0.001). The incidences of early hemorrhages were significantly different between the two groups with 12 in group B (12%) and 2 in group A (1.7%).

**Conclusions:**

Preventive EGD can identify hidden digestive diseases, which may increase the incidence of early hemorrhages.

## Introduction

Atrial Fibrillation (AF) is a common cardiac rhythm disorder with a reported life-time risk of about 25%; it is also an important independent risk factor for cardio embolic ischemic stroke, accounting for at least 15% of all strokes in the USA [1-4]. Cerebrovascular accidents related to AF have a 30-day mortality rate of 25% and are more likely to result in significant disability than noncardioembolic stroke [5]. In non-valvular AF (NVAF), a 4.4% mean incidence of stroke is reported per year; although, the annual stroke rate varies from less than 2% to more than 10% due to patient-specific risks of stroke [2, 3, 6, 7].

Warfarin treatment is recognized worldwide as highly effective in preventing stroke in AF patients, particularly among elderly patients [8]. International studies have shown that warfarin reduces the annual stroke rate in control patients from 4.5% to 1.4%, with a relative risk reduction of 68% [1, 7]. Results from the ACTIVE-W study [9] reaffirm the role of warfarin in stroke prevention for AF patients and strongly suggest the way forward for new agents in this field will not take the form of antiplatelet drugs. However, bleeding is a major complication of both anticoagulant and antithrombotic therapies [9-12]. In randomized clinical trials, the life threatening bleeding rate was 2.3% per year for patients treated with warfarin and 1% in control patients; yet, the incidence changes radically if we consider that gastrointestinal bleeding and major and minor hemorrhage occur in up to 20% of patients treated [9, 10]. A recent, observational cohort study of individuals starting warfarin for the first time highlights the complexity of this issue. In a consecutive series of 472 patients aged 65 years or older, 7% had a major hemorrhage during the first year of warfarin treatment [11]. Further studies are needed to optimize the benefits of “real-world” anticoagulation therapy among patients aged older than 80 years [12].

The risk of major bleeding during warfarin therapy is mainly due to increased age in patients and a history of prior bleeding [11, 13]. From retrospective studies, the most common causes of gastrointestinal bleeding during warfarin treatment are peptic ulcer, erosive gastritis/duodenitis, and neoplasm. The source of bleeding remains undetermined, even after endoscopic investigation, in about one third of patients [14]. Other patient-related factors include hypertension, cerebrovascular disease, heart disease, diabetes, renal failure, alcoholism, liver disease, and concomitant use of antiplatelet drugs [7, 15-17]. The strength of the anticoagulant effect and length of therapy are the major determinants of warfarin-induced bleeding [18, 19].

The aim of the study was to investigate the incidence of digestive hemorrhages in patients with NVAF, scheduled for oral anticoagulant therapy (OAT). The primary outcome of the study was to determine if previous Esophago-Gastro-Duodenoscopy (EGD) in patients with NVAF resulted in a low risk of bleeding during OAT. To test this hypothesis we compared the appearance of digestive hemorrhage in the two groups of patients, those receiving an EGD and those who did not.

## Methods

### Study subjects

We conducted a prospective, population-based observational study on consecutive patients with recurrent paroxysmal, persistent, or permanent NVAF observed at the Emergency and Accident Department of Santa Maria Hospital, Florence, over 24 months, 14 for randomization and 10 for the follow up. Patients were scheduled for OAT, aiming to obtain an international normalized ratio (INR) range of 2 to 3. The value of INR during the study was scheduled at the referring surgery for OAT of the Hospital. Indications for OAT were based on the criteria indicated by the CHADS2 system [2] and only moderate to high risk patients were included. The protocol did not interfere with any programmed or actual antiarrhythmic treatment.

To assess the profile-risk for gastrointestinal bleeding during OAT, patients were evaluated for age, sex, concurrent therapies (i.e., corticosteroids, NSAID, antithrombotics or anticoagulants), alcohol intake, smoking, previous gastrointestinal disorders and/or symptoms suggesting actual gastrointestinal disease (i.e., pre or post-prandial pyrosis), and co-morbidities (i.e., chronic obstructive pulmonary disease (COPD), heart failure, diabetes, and renal failure). Patients with absolute contraindications to OAT such as low compliance, recent surgical intervention, or head trauma and previous intracranial hemorrhage were excluded. The recruitment was undertaken by a team of four researchers, who invited all eligible patients to consider participating in the study. The written informed consent was obtained from each patient. Patients were randomly assigned by computer-generated randomization sequence. This sequence was generated by the Epidemiology Unit of the Health Service, Azienda Sanitaria Fiorentina (Florence, Italy). An upper EGD screening was proposed at the half of patients selected by age, sex, comorbidities and risk factors. Mucosal biopsy to search for Helicobacter Pylori (HP) was done if considered necessary. So, we had two subgroups of patients those who received an EGD and who did not (A and B, respectively).

The small subset of patients who refused EGD was excluded from the analysis. The study was managed by the Tuscany Regional Health Service in line with the European Directive on Medical Research.

Our investigation was carried out with the primary end-point being assessing the presence of upper gastrointestinal lesions at risk of bleeding. The risk level of bleeding was defined by the characteristics of the lesions (Forrest grade or signs of recent bleeding as petechiae). Patients admitted for treatment with OAT were scheduled in a specific register as outpatients to monitor INR and bleeding. On follow up, INR values > 4.5 and the number and kind of drugs took were recorded for patients. The use of a proton pump inhibitor (PPI) or Ranitidine was not allowed after the start of anticoagulant treatment.

During follow-up, we investigated the occurrence of bleeding only from gastrointestinal side. Hemorrhagic events requiring re-hospitalization were first recorded by telephone interview between 10 and 12 months of treatment and, subsequently, by consultation of the medical charts to define the event as a decrease of hemoglobin value of more than 2 g/dl and/or the need to transfuse more than two units of blood. Only these bleedings, defined as major bleedings, were evaluated in follow up.

Hemorrhage, when present, was reported as early (during the first two months) or late (after two months). Patients who died, but did not have hemorrhage, or did not respond to phone interviews were considered lost in the follow up. The Local Ethical Committee approved the study.

### Statistical analysis

Clinical data were recorded in a database. The results were analyzed as percentages; differences between patients with gastrointestinal lesions and controls were calculated by the chi-squared method and values < 0.05 were chosen as significant. Continuous variables are presented as mean ± SD. They were analyzed by Student t-test for unpaired data.

## Results

The study included 268 patients with NVAF (162 males and 106 females) with a mean age of 74 years (range 42 - 86 years). Paroxysmal and persistent AF were found in 90 and 102 patients, respectively, and pharmacological and/or electrical cardioversion was attempted in these patients. The remaining 76 patients had permanent AF that required rate-control pharmacological treatment. Five patients showed signs of gastrointestinal bleeding (hematemesis or melena) and were excluded. Twenty-eight (11%) patients were excluded due to a presumed low compliance to the therapy (i.e., neurological impairment, socio-economic status); two further patients were excluded due to co-morbidities. Three patients did not meet the indications for OAT.

**Figure 1:**
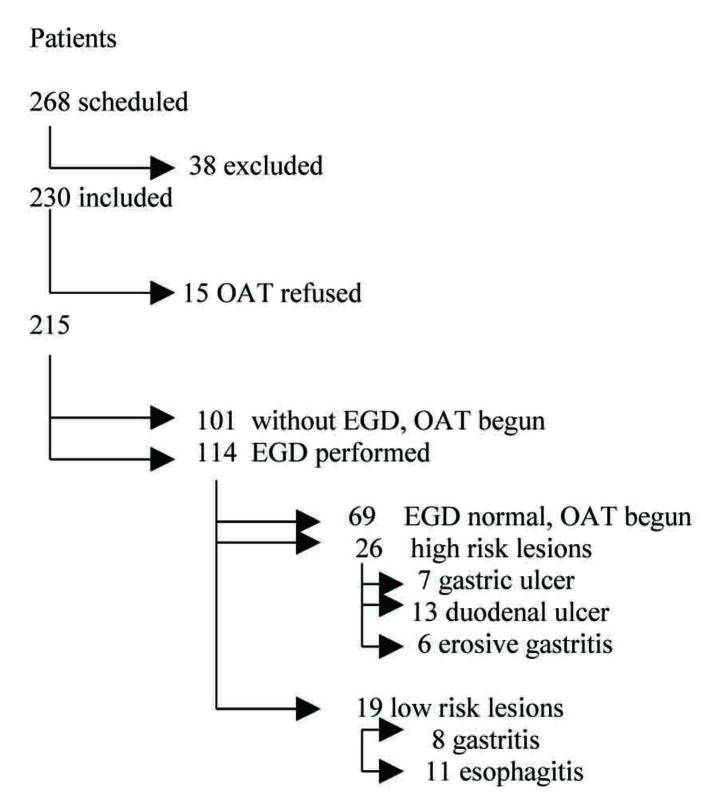
Diagnostic plan.

The remaining 230 patients were stratified as moderate to high risk of stroke as indicated by the CHADS2 scheme [2], and EGD screening and subsequent OAT were proposed. Of the 230 patients, 15 (7%) refused OAT and were excluded; 101 (44%) were scheduled without EGD and began OAT; the remaining 114 patients underwent EGD investigation: 69 patients had negative EGD and began OAT; in 26 patients, high-risk upper gastrointestinal lesions were found (gastric ulcer in 7 patients, duodenal ulcer in 13, and erosive gastritis in 6); and 19 further patients presented with low-risk lesions (8 chronic gastritis and 11 esophagitis, kind A of Los Angeles Classification). Figure 1 depicts the cases and the plan of diagnostic intervention.

All EGD-positive patients underwent medical treatment with PPIs, and subsequent 30-day endoscopic controls showed complete healing in all the patients; OAT was then started. At the end of the randomization two groups of patients underwent follow up: GROUP A: 114 patients who underwent EGD, split into 69 patients (60%) negative by EGD directly submitted to OAT, 26 patients (23%) with high-risk upper gastrointestinal lesions, and 19 patients (17%) with low-risk lesions. Results of fifteen mucosal biopsies to search for HP were available on the 20 patients with ulcer. The HP search was positive only in four cases who underwent protocol of eradication. GROUP B: 101 patients who did not make EGD and began OAT. Data for the two groups of patients are presented in Table 1.

**Table 1:** Patient Characteristics at Randomization and During Follow Up

	Group A	Group B	P
Patient number	114	101	
Age (mean (range))	74 (45 - 86)	77 (42 - 84)	NS*
Males	68	71	NS
INR > 4.5	26	20	NS
NSAIDS or Aspirin or corticosteroids	14	11	NS
Platelet antiaggregants	14	7	NS
Antihypertensive drugs	41	50	NS
Diabetes	7	7	NS
History of cancer	2	1	
Previous digestive diseases or symptoms	6	7	NS
Alcohol or smoking	14	16	NS
Co-morbidities (Renal, COPD, Heart failure, or Coronaropathy)	51	45	NS
β-blockers	79	81	NS
Propafenone/flecainide	30	45	< 0.001
Amiodarone	41	51	< 0.05
Early hemorrhages	2 (1.7%)	12 (12%)	< 0.001
Late hemorrhages	4 (3.5%)	6 (6%)	NS
Lost at follow up	10	12	NS

*NS = not significant

The two groups were comparable for most variables. Significant differences were found between groups for the incidence of antiarrhythmic drugs and for early hemorrhage (P < 0.001). Twenty-two (9%) patients were lost in follow-up; therefore, the incidence of all hemorrhages in the target population was 13%.

## Discussion

Risk stratification scores for gastrointestinal bleeding in AF patients, with indication for OAT, have not yet been established by controlled trials [20, 21]. Although, the occurrence of gastrointestinal bleeding during OAT has been extensively examined in literature, with an incidence of about 20% reported [13, 15]. As the length and strength of OAT have been recognized as major risk factors for bleeding, this explains the high variability in the incidence of gastrointestinal hemorrhage reported. To correctly estimate the true incidence of the event, the frequency by year should be considered.

In randomized clinical trials, the life threatening bleeding rate was 2.3% per year for patients treated with warfarin and 1% in control patients; yet, the incidence changes radically if we consider that gastrointestinal bleeding and major and minor hemorrhage occur in up to 20% of patients treated [9, 10]. A recent, observational cohort study of individuals starting warfarin for the first time highlights the complexity of this issue. In a consecutive series of 472 patients aged 65 years or older, 7% had a major hemorrhage during the first year of warfarin treatment [11]. Further studies are needed to optimize the benefits of “real-world” anticoagulation therapy among patients aged older than 80 years [12].

An incidence of about 2.1% to 3.4% of patients per year was reported in a selected study, using different risk scores [22]. The CHADS2 Score, normally used to define the risk score of cardio-embolism in patients with AF, may be useful in identifying the risk of bleeding too, if restricted to a population over 75 years old [22].

Our study aimed, in the sample population examined, to estimate the risk of bleeding in patients on OAT based on a screening EGD carried out before starting treatment. High-risk upper gastrointestinal lesions were found in 23% of patients who submitted to EGD (group A) and further low-risk lesions were found in 17% of patients. This is in accordance with previous observations [23]. The novel data is that in the two groups, A and B, comparable for age, sex, and risk factors there was a significant difference in incidence of bleeding. Over the follow up, many factors able to induce hemorrhage were present without statistical difference in both groups. These include drugs able to injure gastro-duodenal mucosa, such as NSAIDS or platelets antiaggregants, and the extended INR values. The differences between the two groups in the antiarrhythmics drugs, amiodarone, propafenone and flecainide, are not important to this study because they do not influence the primary end point. Their pharmacological action does not involve the gastro-duodenal mucosa.

The incidences of early hemorrhages were significantly different between the two groups (Table 1) with 12 in group B (12%) and 2 in group A (1.7%). The incidences of all hemorrhages were 18 (18%) in group B vs 6 (7%) in group A at 12 months follow up (Table 1). In literature, there are differences among studies on the incidence of hemorrhage in patients during OAT. Any study is prospective and in two retrospective studies [13, 15], the incidence of digestive hemorrhage at six months was 20% in patients treated without gastric protection. There does not seem to be a difference when gastric protection is given; for in this case, the overall incidence of hemorrhage was 24% [24]. In another study, similar to this one in the number and age of patients [25], two populations of patients on OAT were compared and major bleeding occurred in 12% vs 5.6% at 6 months; it was more common in the usual care group than in the intervention group, in which patients were protected by avoiding gastrolesive drugs.

In our study, the incidence of bleeding was evaluated at two months to point out early bleeding which, in our view, represents the effect of a pre-existing, not identified, digestive lesion. This appears clear by the difference between the two groups (12% vs 1.7% for groups B and A, respectively). At twelve months, the cumulative incidence of hemorrhages was 18% vs 6%. If all hemorrhages are evaluated, the incidence was 13%. Major hemorrhages are defined differently in literature so it is difficult to compare their incidence. The incidence is approximately 2.3% a year so we would expect 5 hemorrhages in 215 patients. Indeed, if we take away the 12 early hemorrhages in group A of this study, we can calculate an incidence of hemorrhage of 5.5%.

An interpretation of the data is that hidden digestive diseases were present in the group that rejected the EGD (group B), which increased the number of early hemorrhages. These data are novel and original and provide an argument “pro” the diagnostic, preventive EGD in subjects with indication for OAT. Our population is at high risk of bleeding because NVAF occurs in an older population. The follow up was completed without PPIs in both groups. Prophylactic use of these drugs can explain the lower incidence of early digestive bleeding in other studies.

### Learning points

This trial provides an argument to suggest preventive Esophago-Gastro-Duodenoscopy in older subjects suffering from atrial fibrillation with indication to oral anticoagulant therapy.
